# Domiciliary monitoring of exhaled nitric oxide in the management of asthma: a pilot study

**DOI:** 10.1186/s12890-024-03031-8

**Published:** 2024-05-17

**Authors:** Hongwen Li, Jiangtao Lin, Qing Zhang, Jingru Wang, Chunxiao Li

**Affiliations:** 1 Department of Geriatric Respiratory Disease, Shandong Provincial Hospital, Affiliated to Shandong First Medical University, Jinan, China; 2 Department of Pulmonary and Critical Care Medicine, China-Japan Friendship Hospital, No 2, East Yinghua Road, Chaoyang District, Beijing, 100029 China; 3 Department of Pulmonary and Critical Care Medicine, The first affiliated hospital of Nanchang University, Nanchang, China; 4 State Key Laboratory of Respiratory Disease, National Clinical Research Center of Respiratory Disease, Guangzhou Institute of Respiratory Health, First Affiliated Hospital of Guangzhou Medical University, Guangdong, China; 5 Peking University China-Japan Friendship School of Clinical Medicine, Beijing, China

**Keywords:** Asthma, Home monitoring, Fe_NO50_, Biomarker, Mobile spirometry

## Abstract

**Background:**

Whether asthma patients could benefit from home monitoring for fractional exhaled nitric oxide (flow of 50 mL/s, Fe_NO50_) is unknown. We explore the application value of home monitoring Fe_NO50_ in daily asthma management.

**Methods:**

Twenty-two untreated, uncontrolled asthma patients were selected. Medical history, blood and sputum samples, pulmonary function, Asthma Control Test (ACT), and other clinical data of the subjects were collected. All subjects underwent daily monitoring for four weeks using a Fe_NO50_ monitor and mobile spirometry (mSpirometry). The diurnal differences and dynamic changes were described. Compare the effect-acting time and the relative plateau of treatment between Fe_NO50_ and mSpirometry monitoring.

**Results:**

In the first two weeks, the morning median (IQR) level of Fe_NO50_ was 44 (35, 56) ppb, which was significantly higher than the evening median level [41 (32, 53) ppb, *P* = 0.028]. The median (IQR) effect-acting time assessed by Fe_NO50_ was 4 (3, 5) days, which was significantly earlier than each measure of mSpirometry (*P* < 0.05). Fe_NO50_ reached the relative plateau significantly earlier than FEV_1_ (15 ± 2 days vs. 21 ± 3 days, *P* < 0.001). After treatment, the daily and weekly variation rates of Fe_NO50_ showed a gradually decreasing trend (*P* < 0.05). The ACT score, sputum eosinophils, and blood eosinophils also significantly improved (*P* ≤ 0.01).

**Conclusions:**

The daily home monitoring of Fe_NO50_ in asthmatic patients showed significant circadian rhythm, and the sensitivity of Fe_NO50_ in evaluating the response to treatment was higher than mSpirometry. The daily and weekly variation rates of Fe_NO50_ change dynamically with time, which may be used to assess the condition of asthma.

## Introduction

Bronchial asthma is a common chronic airway inflammatory disease that causes substantial economic and social burdens [1, 2]. Global Initiative for Asthma (GINA) proposes that the long-term goal of asthma treatment is to achieve symptom control and reduce the risk of acute exacerbations, irreversible airflow limitation, and treatment side effects [3]. At the same time, assessment, adjustment, and monitoring form a continuous cycle in asthma treatment and management strategy. Thus, effective self-assessment and monitoring are vital for asthma patients to achieve long-term treatment goals [4]. However, previous studies have shown that the natural history of asthma is heterogeneous and complex, characterized by circadian rhythms and long-term dynamic changes [5]. Therefore, daily asthma management should include dynamic changes, not just the absolute value of a single measurement.

Studies have shown inconsistencies in symptoms, lung function, and airway inflammation of asthma patients [6]. Self-management based on symptoms and peak expiratory flow (PEF) may still miss patients at risk of severe acute exacerbations in the future [7, 8]. There are also inconsistencies between physician and patient in the assessment of asthma control [9]. So, perhaps more methods are needed for daily asthma assessment. Fractional exhaled nitric oxide (flow of 50 mL/s, Fe_NO50_) is one of the tools to assess airway inflammation, which is non-invasive, simple, rapid, and is currently mainly used in hospital scenarios [10]. Studies have shown that increasing the number and frequency of Fe_NO50_ monitoring helps predict asthma control status [11]. The daily fluctuation of Fe_NO50_ with different asthma control statuses is different [12]. Studying the long-term variation pattern of Fe_NO50_ measurements makes it possible to identify the risk of acute exacerbations [13–15]. Simultaneous daily monitoring of Fe_NO50_ can also assess compliance [10, 16, 17] and responsiveness to inhaled corticosteroids (ICS) for Type 2 asthma [18–21]. In summary, applying Fe_NO50_ in daily asthma self-monitoring may be significant for asthma management.

More convenient products are applied to self-monitor chronic diseases with the development of science and technology and improved economic levels [7]. The emergence of daily home monitoring devices and innovative applications has made it possible to monitor circadian rhythms and daily changes for asthma patients. However, whether asthma patients could benefit from it is not fully known due to the lack of prognostic data [22]. We attempted to analyze the pattern of longitudinal dynamic changes in Fe_NO50_ after treatment in uncontrolled asthmatic patients who were not regularly treated. To initially explore the value of Fe_NO50_ domiciliary monitoring in the daily management of asthma.

## Methods

### Subjects

Twenty-two asthma patients who visited the respiratory outpatient of China-Japan Friendship Hospital from October 2019 to December 2021 were prospectively included.

Inclusion criteria: (1) age ≥ 18 years old; (2) fulfilled the diagnostic criteria of bronchial asthma defined by GINA 2018 [3]; (3) asthma symptom control was assessed as uncontrolled according to GINA2018 [3].

Exclusion criteria: (1) subjects underwent other interventional clinical trials 30 days before enrollment; (2) subjects with other pulmonary diseases or other severe system diseases that may affect the conduct of the study; (3) subjects with a smoking index > 10 pack-years and a history of smoking for nearly one year; (4) subjects had been on regular asthma therapy within 12 weeks before enrollment; (5) subjects had respiratory tract infection within four weeks before enrollment.

Written informed consent was obtained from each participant. The China-Japan Friendship Hospital ethics committee approved this study (No. 2018-19-k14, approval date: February 6^th^, 2018).

### Study design

Enrollment stage: All subjects performed the asthma control test (ACT) [23], mini-asthma quality of life questionnaire (mini-AQLQ) [24], differential blood count, serum total IgE (enzymatic chemiluminescence, Beckmen Coulter, USA), spirometry, Fe_NO50_ (NIOX Vero, Circassia (Beijing) Medical Device Co., Beijing, China), differential induced sputum count. Mobile spirometry (A1, Breath Home, China) [25] and Fe_NO50_ monitor (NIOX Vero, Circassia (Beijing) Medical Device Co., Beijing, China) were provided to each subject to measure Fe_NO50_ and mSpirometry twice a day over four weeks at home. On the day of enrollment, subjects were trained on using the equipment mentioned above (viewing usage videos and on-site instruction). Subjects were contacted during subsequent use to ensure they correctly mastered the usage methods.

The treatment strategy is not affected by the study, and the patient's treatment plan is formulated by the physician, with medication recommendations based on the GINA 2018 guidelines [3].

Domiciliary monitor stage: Subjects were asked to measure Fe_NO50_ and mSpirometry twice daily during the same period, between 06:00 to 08:00 and 20:00 to 22:00, respectively. Before medication, Fe_NO50_ measurement was first taken, and the results were automatically recorded on the monitor. When the subjects returned the device, all measurements were transmitted to the computer. Mobile spirometry was taken three times, and the best of the three readings were automatically uploaded to an electronic diary card. Peak expiration flow (PEF), forced expiratory volume in 1 second (FEV_1_), forced vital capacity (FVC), the maximum expiratory flow rate at 75%/50%/25% of the vital capacity (MEF_75_, MEF_50_, and MEF_25_) were collected by mSpirometry.

End of follow-up: ACT, mini-AQLQ, spirometry, Fe_NO50_, differential blood, and induced sputum count were reviewed again.

### Statistical Analysis

The normal distribution data were represented by mean ± standard deviation (sd). Non-normally distributed data were shown as median (interquartile range, IQR). A comparison of each measure before and after follow-up was performed using paired samples t-test and Wilcoxon signed-rank test. The categorical variables were expressed by frequency (composition ratio or percentage) and compared by the chi-square test.

Indicators and calculation formulas representing the variation of Fe_NO50_ and mSpirometry: diurnal variation rate = (highest in a day - lowest in a day)/ (mean of highest versus lowest in a day)×100; mean daily variation rate = mean of diurnal variation rate over 1week; weekly variation rate = (highest over two weeks - lowest over two weeks)/ (mean of highest versus lowest over two weeks)×100.

A repeated-measures analysis of variance was used to compare diurnal differences in Fe_NO50_ logarithmic transformed values and mSpirometry. The least-square method was used to perform curve fitting for each subject's daily monitoring results of Fe_NO50_ and mSpirometry, and the second derivative was used to calculate the inflection point. The inflection point in this study was the transition point for the improvement of Fe_NO50_ and mSpirometry, and its progress slowed down after the inflection point. The relative plateau of treatment was defined as the time to reach the inflection point [26]. The effect-acting time was calculated with a Fe_NO50_ reduction of more than 20% and an improvement in mSpirometry of more than 10% as criteria [10, 27]. ANOVA and Friedman's test were used to compare the relative plateau and the effect-acting of treatment between Fe_NO50_ and mSpirometry. Friedman's test was also used to compare the differences in variation rates of Fe_NO50_ and mSpirometry. A two-tailed *p*-value of < 0.05 was considered significant. All statistical analyses were performed with SPSS 20 (IBM-SPSS, Armonk, NY, USA) and Matlab software (Mathworks, Inc., Natick, MA, USA).

## Results

### Characteristics of subjects

A total of 22 subjects were included in the study, with 40 ± 14 years old (range 18-64 years). There were slightly more female subjects (*n* = 14, 63.6%), and the BMI was 23.74 ± 4.60 kg/m^2^. The median (IQR) duration of illness was 2.75 (0.84, 8.87) years. The main co-morbidities of the subjects were allergic rhinitis (90.9%), nasal polyps (18.2%), and eczema (22.7%). A history of allergy was present in 59.1% of the subjects, and the median (IQR) serum total IgE level was 185.50 (86.88, 470.75) IU/ml. All subjects received salmeterol/fluticasone (50/250 μg) twice daily, with three subjects receiving montelukast and three subjects receiving tiotropium.

The effective Fe_NO50_ and mSpirometry monitoring were 1035 and 991 times, respectively, and the overall adherence rates were 84.0% (1035/1232) and 80.4% (991/1232), respectively. Of the enrolled subjects, F_eNO50_ baseline level ≥ 50 ppb in 19 (86.4%) patients; sputum eosinophil baseline level ≥ 3% in 21 (95.4%) patients; and 16 (72.7%) patients had blood eosinophils baseline level ≥ 300/μL; 15 patients (68.2%) had FEV_1_% baseline level > 80%.

### Assessment of the disease condition before and after treatment

After four weeks of treatment, the subjects showed significant improvement in all measurements (Table 1). A significant improvement in the ACT (improvement of more than 3 points) [23] was observed in 17 subjects, with an ACT score ranging from 16 ± 4 points improved to 23 ± 2 points (*P* < 0.001). In mini-AQLQ, overall score, symptoms, activity, emotion, and environmental scores improved significantly (*P* < 0.01). The pulmonary function was significantly improved (*P* ≤ 0.01). In terms of inflammation, either Fe_NO50_ or sputum and blood eosinophils were significantly reduced (*P* < 0.01) (Table 1).

**Table 1:** Assessment of the disease condition before and after treatment

	baseline	visit	*P*
ACT (mean ± SD)	16 ± 4	23 ± 2	<0.001
mini-AQLQ (mean ± SD)			
Symptoms	3.84 ± 0.98	5.63 ± 1.22	<0.001
Activity	4.93 ± 1.27	6.01 ± 0.94	0.001
Emotion	3.71 ± 1.34	5.03 ± 1.49	0.002
Environment	3.67 ± 1.49	5.00 ± 1.09	0.001
Overall score	4.06 ± 0.95	5.49 ± 1.04	0.001
Spirometry function			
FEV_1_ (L, mean ± SD)	2.47 ± 0.76	2.89 ± 0.60	0.002
FEV_1_% (%, mean ± SD)	81.2 ± 20.9	95.9 ± 10.5	<0.001
FEV_1_/FVC (%, mean ± SD)	66.4 ± 14.3	72.2 ± 7.8	0.002
PEF (L, mean ± SD)	5.72 ± 1.79	7.27 ± 1.39	<0.001
MMEF_75/25_ (L, mean ± SD)	1.74 ± 0.88	2.15 ± 0.79	0.003
MEF_75_ (L, mean ± SD)	4.00 ± 1.76	5.30 ± 1.26	<0.001
MEF_50_ (L, mean ± SD)	2.06 ± 0.97	2.58 ± 0.81	0.002
MEF_25_ (L, mean ± SD)	0.79 ± 0.44	0.86 ± 0.44	0.026
Fe_NO50_ [ppb, median (IQR)]	80 (56, 117)	27 (18, 47)	<0.001
Sputum eosinophils [%, median (IQR)]	25.8 (15.0, 59.6)	2.8 (1.0, 14.0)	0.004
Blood eosinophils [cells/μL, median (IQR)]	380 (283, 658)	255 (188, 280)	0.001

*ACT* asthma control test, *mini-AQLQ* mini-asthma quality of life questionnaire, *FEV*_1_ forced expiratory volume in 1-second, *FVC* forced vital capacity, *PEF* peak expiratory flow, *MMEF*_75/25_ maximum mid expiratory flow, *MEF*_75_/ *MEF*_50_/ *MEF*_25_ maximum expiratory flow rate at 75%/ 50%/ 25% of the vital capacity, *Fe*_NO50_ fractional exhaled nitric oxide (flow of 50 mL/s)

### *Diurnal variation of Fe*_*NO50*_* and mSpirometry*

**Fig. 1:**
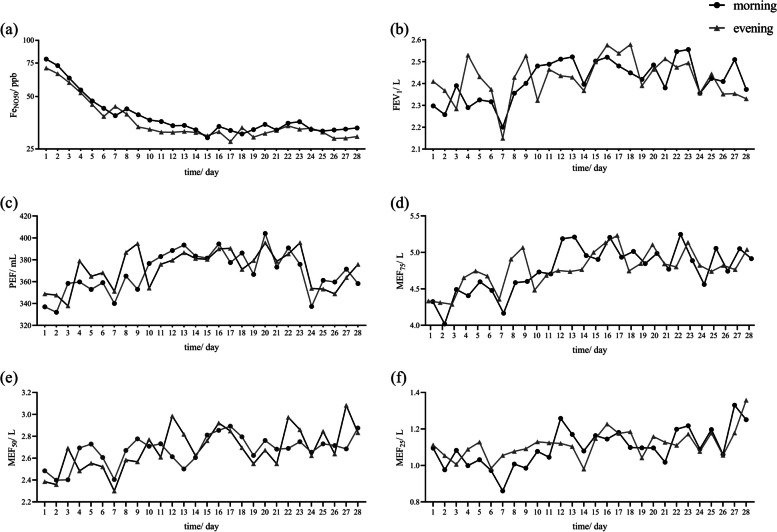
Diurnal variation curves of domiciliary monitoring. **a** Fe_NO50_ diurnal variation curves, **b** FEV_1_ diurnal variation curves, **c** PEF diurnal variation curves, **d** MEF_75_ diurnal variation curves, **e** MEF_50_ diurnal variation curves, **f** MEF_25_ diurnal variation curves. Fe_NO50_: fractional exhaled nitric oxide (flow of 50 mL/s), FEV_1_: forced expiratory volume in 1-second, PEF: peak expiratory flow, MEF_75_/ MEF_50_/ MEF_25_: maximum expiratory flow rate at 75%/ 50%/ 25% of the vital capacity

There was a significant diurnal difference in Fe_NO50_ daily monitoring in the first two weeks. The morning median (IQR) level of Fe_NO50_ was 44 (35, 56) ppb, which was significantly higher than the evening median level [41 (32, 53) ppb, *P* = 0.03]. However, after two weeks of treatment, the significant difference between day and night disappeared (*P* = 0.17). In our study, no significant differences were found in any indicators of mSpirometry between day and night (Fig. 1).

### *Dynamic fluctuation of Fe*_*NO50*_* and mSpirometry*

**Fig. 2:**
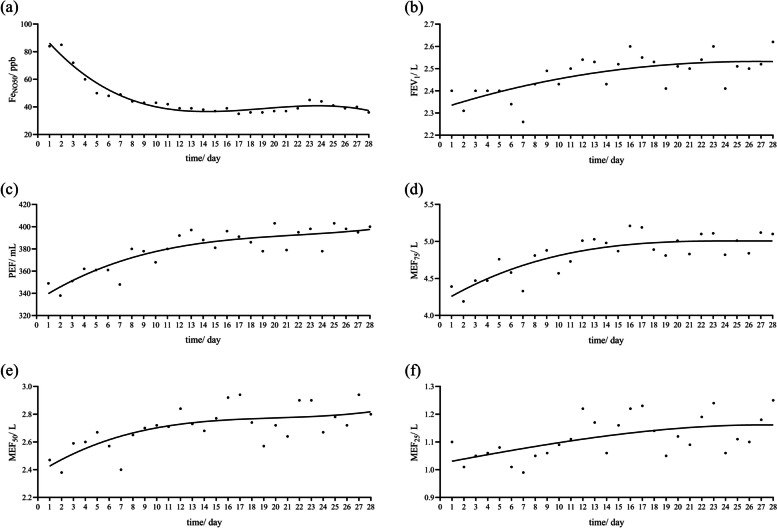
Fitting curve of domiciliary monitoring. **a** Fitting curve of Fe_NO50_ (*R*^*2*^ = 0.698 ± 0.239), **b** Fitting curve of FEV_1_ (*R*^*2*^ = 0.476 ± 0.270), **c** Fitting curve of PEF (*R*^*2*^ = 0.564 ± 0.220), **d** Fitting curve of MEF_75_ (*R*^*2*^ = 0.499 ± 0.203), **e** Fitting curve of MEF_50_ (*R*^*2*^ = 0.503 ± 0.237), **f** Fitting curve of MEF_25_ (*R*^*2*^ = 0.449 ± 0.158). Fe_NO50_: fractional exhaled nitric oxide (flow of 50 mL/s), FEV_1_: forced expiratory volume in 1 second, PEF: peak expiratory flow, MEF_75_/ MEF_50_/ MEF_25_: maximum expiratory flow rate at 75%/ 50%/ 25% of the vital capacity, *R*^*2*^: coefficient of determination

The best curve fit of the daily monitoring indices of Fe_NO50_ and mSpirometry for the subject is shown in Fig. 2. In general, Fe_NO50_ and Spirometry improved gradually over time with treatment. The median (IQR) effect-acting time assessed by Fe_NO50_ was 4 (3, 5) days, which was significantly earlier than mSpirometry (*P* < 0.05) (Table 2). After Bonferroni correction, Fe_NO50_ reached the relative plateau significantly earlier than FEV_1_ (15 ± 2 days vs. 21 ± 3 days, *P* < 0.001), but there was no statistical difference with other indicators of mSpirometry (Table 2).

**Table 2:** Exhaled nitric oxide fraction and mobile spirometry assessment therapeutic effect

	effect-acting time^†^ [day, median (IQR)]	*P*^‡^	relative plateau time^§^ [day, mean ± SD)]	*P*^‡^
Fe_NO50_	4 (3, 5)		15 ± 2	
FEV_1_	11 (4, 28)	0.001	21 ± 3	<0.001
PEF	8 (4, 20)	0.005	18 ± 3	0.013
MEF_75_	11 (4, 27)	0.001	14 ± 4	0.754
MEF_50_	8 (4, 16)	0.009	12 ± 4	0.012
MEF_25_	12 (4, 28)	<0.001	12 ± 3	0.011

^†^The effect-acting time was calculated with Fe_NO50_ reduction of more than 20% and mSpirometry improvement of more than 10% as criteria

^‡^Fe_NO50_ vs mSpirometry (PEF, FEV_1_, MEF_75_, MEF_50_, MEF_25_)

^§^The relative plateau time in this study was the transition point for improvement of Fe_NO50_ and mSpirometry, and its progress slowed down after the transition point.* Fe*_NO50_: fractional exhaled nitric oxide (flow of 50 mL/s), *mSpirometry*: mobile spirometry, *PEF*: peak expiratory flow, *FEV*_1_: forced expiratory volume in 1-second, *MEF*_75_/ *MEF*_50_/ *MEF*_25_: maximum expiratory flow rate at 75%/ 50%/ 25% of the vital capacity

### *Variation rates of Fe*_*NO50*_* and mSpirometry*

**Fig. 3:**
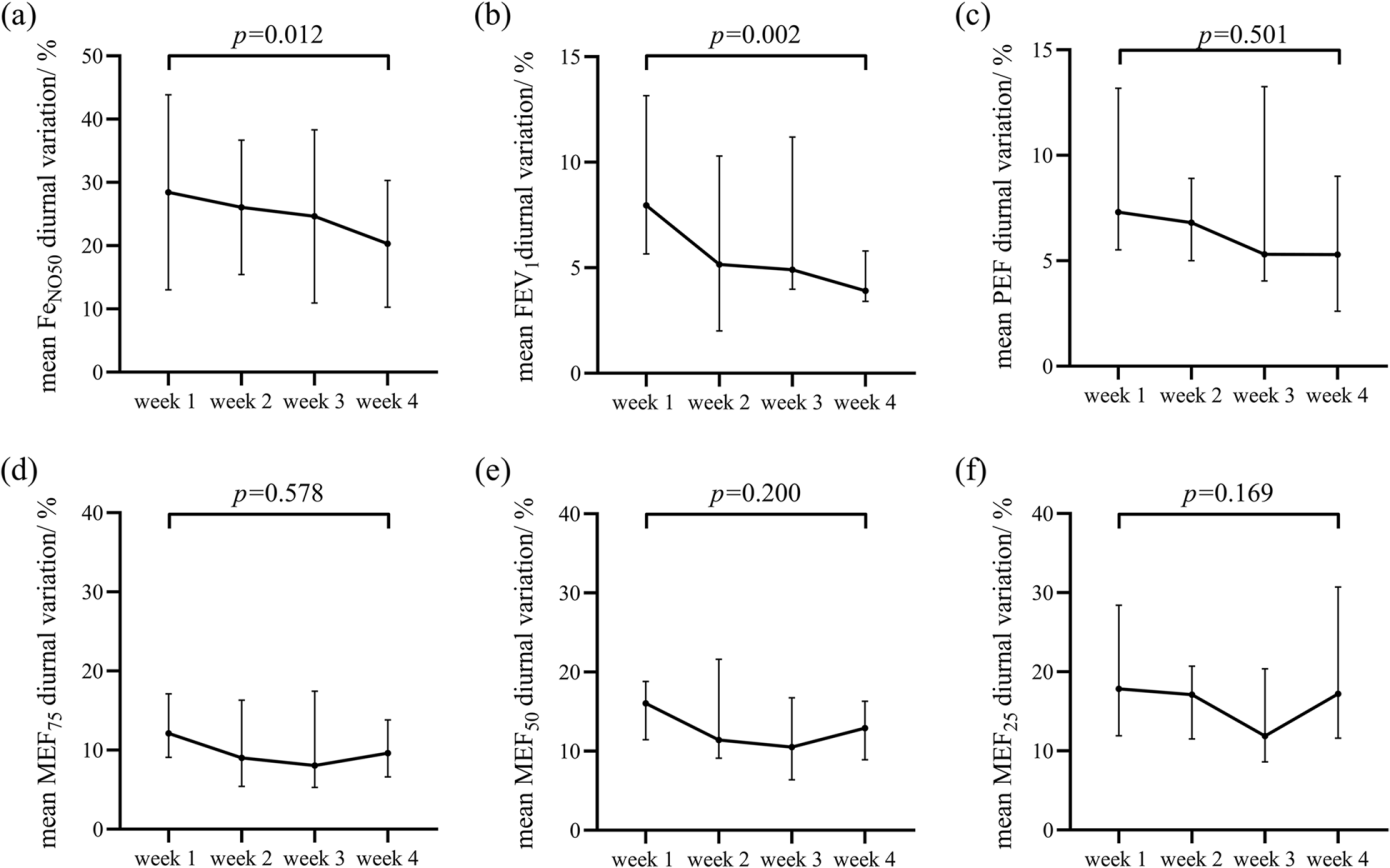
Daily variation rate curves of domiciliary monitoring. **a** Daily variation rate curves of Fe_NO50_, **b** Daily variation rate curves of FEV_1_, **c** Daily variation rate curves of PEF, **d** Daily variation rate curves of MEF_75_, **e** Daily variation rate curves of MEF_50_, **f** Daily variation rate curves of MEF_25_. Fe_NO50_: fractional exhaled nitric oxide (flow of 50 mL/s), FEV_1_ forced expiratory volume in 1 second, PEF: peak expiratory flow, MEF_75_/ MEF_50_/ MEF_25_: maximum expiratory flow rate at 75%/ 50%/ 25% of the vital capacity

**Fig. 4:**
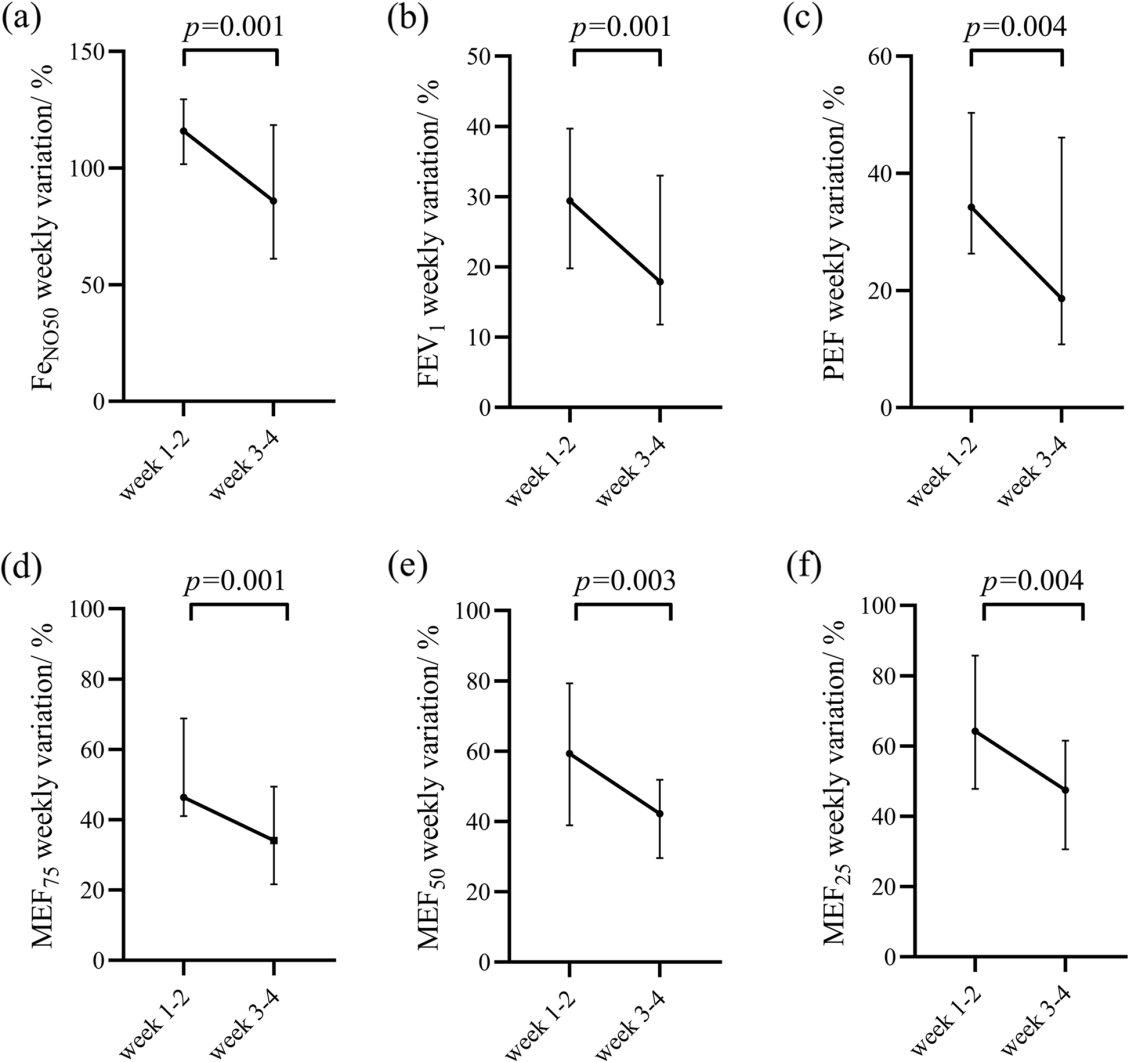
Weekly variation rates of domiciliary monitoring. **a** Weekly variation rate of Fe_NO50_, **b** Weekly variation rate of FEV_1_, **c** Weekly variation rate of PEF, **d** Weekly variation rate of MEF_75_, **e** Weekly variation rate of MEF_50_, **f** Weekly variation rate of MEF_25_. Fe_NO50_ fractional exhaled nitric oxide (flow of 50 mL/s), FEV_1_: forced expiratory volume in 1-second, PEF: peak expiratory flow, MEF_75_/ MEF_50_/ MEF_25_: maximum expiratory flow rate at 75%/ 50%/ 25% of the vital capacity

After ICS treatment, the daily variation rates of Fe_NO50_ and mSpirometry showed a gradually decreasing trend. The average daily variation rates of Fe_NO50_ and FEV_1_ at week 1 were significantly higher than those at week 4 (*P* < 0.05), while there was no significant difference in PEF, MEF_75_, MEF_50_, and MEF_25_. (Fig. 3). There was a substantial reduction in weekly variation rates of Fe_NO50_ and mSpirometry among the participants (*P* < 0.05) (Fig. 4).

## Discussion

Through daily domiciliary monitoring of untreated, uncontrolled asthmatic patients, we found significant diurnal differences and daily dynamic changes in Fe_NO50_ and mSpirometry, which can be used to evaluate asthma patients' response to treatment and condition assessment.

Fe_NO50_ is a sensitive biomarker that reflects the inflammation of airway eosinophils [28]. In the early stage of treatment, Fe_NO50_ was significantly higher in the morning than at night in asthmatic patients, consistent with previous findings [21, 29]. Although studies have shown that the decrease of FEV_1_ affects the results of Fe_NO50_, the effect of the change in airway diameter on Fe_NO50_ will be offset when the airway inflammation is higher [30, 31]. However, the disappearance of diurnal differences in asthmatic patients after treatment may be related to airway inflammation and lung function improvement [32, 33].

The primary purpose of this study is to observe the impact of home monitoring on evaluating treatment outcomes. After four weeks of treatment, all participants showed improvement in symptoms and inflammation levels. Therefore, although different treatment plans may cause biases, we believe the impact on the conclusion is relatively tiny.

Peak-trough times of lung function and biomarkers in asthma patients may differ due to individual chronotype differences [34]. Lung function fluctuates between 6-7 days in Fig. 1, but there is no significant difference. Although the measurement period was specified to avoid bias, the personal measurement time or additional measurement period was not selected according to the living habits, thus reducing the reliability of dynamic variation. No significant diurnal differences in lung function were found in this study, which may be related to the relatively good lung function of the included subjects, reducing the sensitivity of diurnal differences.

We characterized the changing trend of Fe_NO50_ in treated Type 2 asthmatic patients. We found that Fe_NO50_ could detect the treatment response for 3-5 days and reached a relative plateau for two weeks. Various indices (PEF, FEV1, MEF_75_, MEF_50_, MEF_25_) of mSpirometry improved gradually, with treatment effect-acting time around 8~12 days and reached a relative plateau of improvement around 2-3 weeks [26, 35]. However, due to the relatively low goodness of fit of the fitting curves for mSpirometry in this study, the treatment turning point of mSpirometry still needs further validation with extensive sample data.

Daily monitoring found that the treatment effect assessed by Fe_NO50_ was significantly earlier than mSpirometry, and the time to reach the relative plateau of treatment was substantially earlier than FEV_1_. As we can see, Fe_NO50_ was more sensitive than mSpirometry in assessing responsiveness to asthma therapy. Meanwhile, the latest research indicates that Fe_NO50_ is a risk biomarker identifying patients at increased risk of lung function decline [36].

After treatment, the daily and weekly variation rates of Fe_NO50_ and mSpirometry showed a decreasing trend. Our study found that the average daily variation of Fe_NO50_ in the first week was significantly higher than in the fourth week. Therefore, the improvement of diurnal variation in Fe_NO50_ can also be used to evaluate the effectiveness of treatment in asthma. This variability over time suggests that the domiciliary monitoring strategy has the advantage of detecting daily and long-term changes in physiology and inflammation, providing substantial evidence to predict future exacerbations and disease assessment. After four weeks of treatment, the participants showed significant improvements in asthma control, quality of life, lung function, and inflammation. With the considerable improvement of symptoms and clinical indicators, the variation rate of domiciliary monitors gradually decreased, which is an essential clinical signal to evaluate the changes in the conditions of asthma patients.

The study had limitations. Firstly, as our study is a pilot study, all admitted patients were untreated type 2 asthma. We did not include patients with severe asthma. Still, previous studies have shown that Fe_NO50_ can also serve as an inflammatory marker for evaluating treatment response in severe asthma populations. [37]. A large sample study was needed to demonstrate the general generalization of the variation pattern. Secondly, complex device use, frequency of daily readings, and strict monitoring times reduce the completion of measures [38, 39]. Still, previous studies have also shown that remote monitoring devices allow subjects to understand their self-control levels and improve patient compliance [40, 41]. Although there was no monitoring of drug adherence, the completion rate of this study exceeded 80%, indirectly indicating that drug adherence is still acceptable. Thirdly, our study could not compare mSpirometry A1 with the lung function laboratory device. The study found that only a portion of mSpirometry A1 (BreathHome, China) met the quality and performance evaluation standards [25]. However, considering this study mainly observed dynamic changes, the research results are still acceptable.

In conclusion, this pilot study of domiciliary monitoring found that Fe_NO50_ in uncontrolled asthma patients has significant diurnal differences and is superior to mSpirometry in assessing sensitivity to treatment response. The dynamic fluctuation of Fe_NO50_ may be available for evaluating disease conditions in asthmatics. Studies with large samples and long observation periods are expected to explore mobile domiciliary monitors in asthma management.

## Data Availability

The datasets used and analyzed during the current study are available from the corresponding author upon reasonable request.

## Abbreviations

*ACT*: Asthma control test
*BMI*: Body mass index
*Fe*_*NO50*_: Fractional exhaled nitric oxide (flow of 50 mL/s)
*FEV*_*1*_: Forced expiratory volume in 1 second
*FVC*: Forced vital capacity
*GINA*: Global initiative for asthma
*ICS*: inhaled corticosteroids
*IQR*: interquartile range
*MEF*_*75*_: Maximum expiratory flow rate at 75% of the vital capacity
*MEF*_*50*_: Maximum expiratory flow rate at 50% of the vital capacity
*MEF*_*25*_: Maximum expiratory flow rate at 25% of the vital capacity
*mini-AQLQ*: mini Asthma Quality of Life Questionnaire
*mSpirometry*: mobile spirometry
*PEF*: peak expiratory flow
